# Forage Height and Above-Ground Biomass Estimation by Comparing UAV-Based Multispectral and RGB Imagery

**DOI:** 10.3390/s24175794

**Published:** 2024-09-06

**Authors:** Hongquan Wang, Keshav D. Singh, Hari P. Poudel, Manoj Natarajan, Prabahar Ravichandran, Brandon Eisenreich

**Affiliations:** Lethbridge Research and Development Centre, Agriculture and Agri-Food Canada (AAFC), 5403 1st Avenue South, Lethbridge, AB T1J 4B1, Canada; hongquan.wang@agr.gc.ca (H.W.); hari.poudel@agr.gc.ca (H.P.P.); manoj.natarajan@agr.gc.ca (M.N.); prabahar.ravichandran@agr.gc.ca (P.R.); brandon.eisenreich@agr.gc.ca (B.E.)

**Keywords:** forage, canopy height, above-ground biomass, UAV, multispectral, RGB, sensor selection

## Abstract

Crop height and biomass are the two important phenotyping traits to screen forage population types at local and regional scales. This study aims to compare the performances of multispectral and RGB sensors onboard drones for quantitative retrievals of forage crop height and biomass at very high resolution. We acquired the unmanned aerial vehicle (UAV) multispectral images (MSIs) at 1.67 cm spatial resolution and visible data (RGB) at 0.31 cm resolution and measured the forage height and above-ground biomass over the alfalfa (*Medicago sativa* L.) breeding trials in the Canadian Prairies. (1) For height estimation, the digital surface model (DSM) and digital terrain model (DTM) were extracted from MSI and RGB data, respectively. As the resolution of the DTM is five times less than that of the DSM, we applied an aggregation algorithm to the DSM to constrain the same spatial resolution between DSM and DTM. The difference between DSM and DTM was computed as the canopy height model (CHM), which was at 8.35 cm and 1.55 cm for MSI and RGB data, respectively. (2) For biomass estimation, the normalized difference vegetation index (NDVI) from MSI data and excess green (ExG) index from RGB data were analyzed and regressed in terms of ground measurements, leading to empirical models. The results indicate better performance of MSI for above-ground biomass (AGB) retrievals at 1.67 cm resolution and better performance of RGB data for canopy height retrievals at 1.55 cm. Although the retrieved height was well correlated with the ground measurements, a significant underestimation was observed. Thus, we developed a bias correction function to match the retrieval with the ground measurements. This study provides insight into the optimal selection of sensor for specific targeted vegetation growth traits in a forage crop.

## 1. Introduction

Plant height and biomass are two essential biophysical parameters to quantify forage growth conditions and yield. The accurate estimation of forage height and biomass holds significant importance for plant breeders in making selection decisions, livestock management, pasture productivity assessment, and ecological monitoring. The traditional approaches for measuring crop physical parameters are destructive, time-consuming, labor-intensive, and highly subjective. With the development of smart sensors and image processing approaches, drone-based remote sensing provides a powerful tool to efficiently monitor forage growth at various temporal and spatial scales [1,2].

Compared to satellite platforms, drone-based multispectral imaging (MSI) systems are more flexible to monitor a landscape with designed imaging parameters such as flight altitude, speed, and observation angles [3]. For the data collected by low-altitude systems, the atmospheric influences on the images are also reduced, thus avoiding complex atmospheric corrections [4]. Furthermore, owing to very high spatial resolution in centimeters, UAV observation data are widely used in precision agriculture applications such as disease detection, crop growth monitoring, and yield prediction. On the other hand, the drone system is not appropriate for applications over vast areas, where satellite remote sensing is powerful. The acquisition of drone data is also expensive and time-consuming in contrast with satellite data access. For temporal analysis, it is challenging to maintain consistent solar radiation conditions across a time series of drone data.

Several approaches have been employed for forage height estimation using UAV data [5,6]. Structure from motion (SfM) photogrammetry [7] reconstructs 3D models from overlapping UAV images, allowing for precise measurement of forage height. Integration of RGB imagery with SfM facilitates detailed surface modeling, capturing variations in terrain and vegetation height. LiDAR sensors mounted on UAVs provide direct measurements of vegetation height by emitting laser pulses and analyzing the reflected signals. Coupled with MSI data, LiDAR enables accurate estimation of vegetation height even in complex terrain conditions [8]. Recently, the machine learning algorithms trained on UAV-derived MSI and RGB data have shown promise in predicting forage height [9]. Supervised learning models, such as random forest and support vector regression, utilize spectral and textural features extracted from imagery to infer forage height with high accuracy [10].

Accurate estimation of forage biomass is essential for optimizing livestock grazing, monitoring pasture productivity, and assessing ecosystem health. UAV-based MSI and RGB data offer valuable insights into vegetation structure and biochemical composition, enabling robust biomass estimation models. The spectral indices are commonly used to estimate the forage biomass [11]. The MSI allows for the calculation of vegetation indices, such as NDVI (normalized difference vegetation index) and NDRE (normalized difference red edge), which correlate with biomass density [12]. Integration of spectral indices with machine learning algorithms enhances the accuracy of biomass prediction models [13]. Additionally, integrating MSI and RGB imagery enables comprehensive characterization of vegetation properties, including biomass [14]. Fusion techniques combine spectral information from MSI sensors with spatial details from RGB imagery, enhancing the discrimination of biomass variations within a landscape [15].

MSI and RGB sensors provide valuable data for vegetation analysis. Multispectral sensors capture information across specific spectral bands, enabling the discrimination of vegetation types, health status, and biomass estimation. On the other hand, an RGB sensor offers high-resolution imagery for detailed feature extraction and analysis. This study evaluates the effectiveness of UAV-based MSI and RGB imaging systems to characterize Alfalfa (*Medicago sativa* L.) canopy height and above-ground biomass (AGB) proxy for efficient phenotypic traits estimation and cultivar selections [16]. The finding in this paper contributes to suggesting different sensors such as MSI and RGB for the UAV system to monitor forage biomass and height. We aim to identify the optimal sensor for different crop growth parameters.

## 2. Data and Methods

### 2.1. Study Site and Data Acquisition

The study site is located at an experimental farm of Agriculture and Agri-Food Canada, situated within Lethbridge Research and Development Center in Southern Alberta, Canada (49°42′31.4″ N, 112°45′34.7″ W).

We designed three randomized blocks, each of which had a total of 98 alfalfa plots planted (294 plots for three blocks). For each block, we set a 1 m distance between plots, with each plot containing 20 plants spaced 0.33 m apart. For each plot with an area of 6.27 m^2^, the biomass was harvested using a customized harvester on 8 August and 12 September 2022, respectively. The alfalfa height was measured using a standard ruler by randomly taking 3 samples over each plot on 12 October 2022.

**Figure 1:**
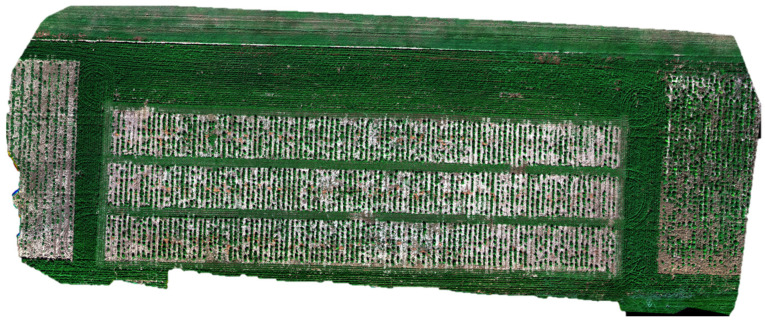
Study site with UAV multispectral imagery on 13 October 2022.

Over the study site on 13 October 2022, we acquired the UAV-based MSI and RGB images [17] at 25 m flight altitude, using the Micasense (Seattle, MA, USA) RedEdge-P MSI sensor (6 bands) and DJI (Shenzhen, China) Zenmuse P1 camera (45MP RGB), respectively. The UAV flights were set with 85% and 80% for forward and side overlaps to enhance the quality and accuracy of the orthomosaic. Although there was a temporal gap between the UAV data acquisition and ground measurements of biomass, this study was designed to analyze the sensitivity of UAV MSI and RGB-derived features to ground measurements of vegetation parameters including canopy height and above-ground biomass. The height measurements were matched with the UAV data acquisitions. The original data were processed using the commercial photogrammetry software (Pix4D Mapper 4.8.4) to implement radiometric calibration and image stitching to obtain the orthomosaic map of the alfalfa experimental trial in Figure 1. Owing to the high spatial resolution, we could capture every single plot.

### 2.2. Methods

**Figure 2:**
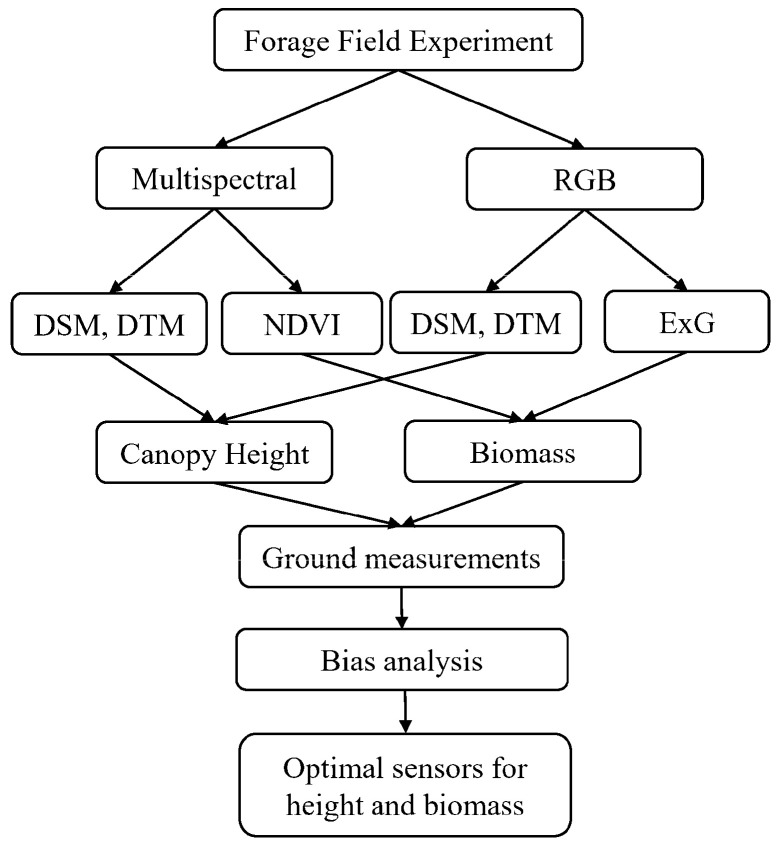
UAV multispectral and RGB imaging analysis workflow to estimate canopy height and above-ground biomass.

This study aims to estimate the above-ground biomass and canopy height for forage crops (Figure 2). We compared the performance between MSI and RGB sensors to retrieve the height and biomass information. Finally, optical sensors were suggested to measure canopy height and forage biomass for practical applications.

#### 2.2.1. Canopy Height Estimation

**Figure 3:**
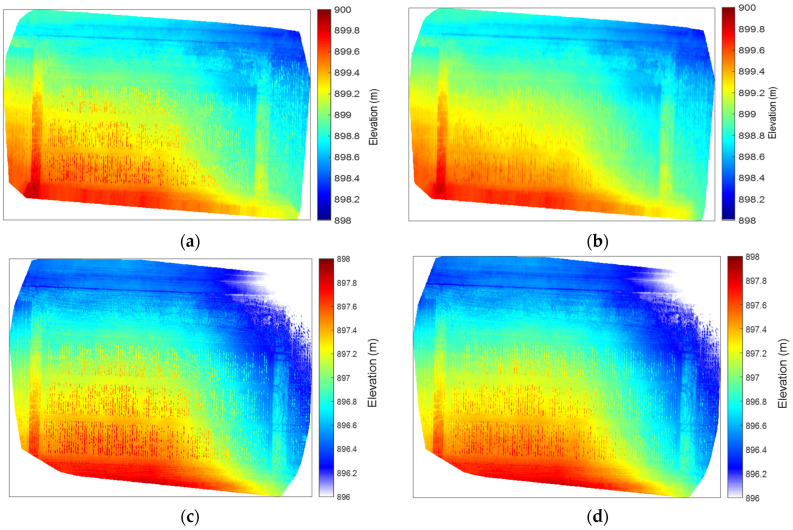
(**a**) DSM and (**b**) DTM from multispectral imagery, in comparison with (**c**) DSM and (**d**) DTM from RGB imagery.

Based on the orthomosaic, we generated a digital surface model (DSM), as seen in Figure 3a, at a fine spatial resolution of 1.67 cm/pixel from the MSI data using structure from motion algorithms [18,19]. This algorithm can estimate the 3D structure of a scenario from a series of 2D images [20]. In order to estimate canopy height, we also calculated the digital terrain model (DTM). However, the spatial resolution of the digital terrain model (DTM) in Figure 3b was degraded to 8.35 cm/pixel, five times lower than that of the DSM [21]. The degradation of spatial resolution impacts the performance of the crop height estimation. In addition, the higher vegetation conditions reduced the quality of the DTM, as fewer ground pixels are available to reconstruct surface terrain under the vegetation cover [22]. This leads to slight remaining effects of alfalfa planting structure in the DTM in Figure 3b.

In comparison with the MSI data, the RGB data were acquired at a higher spatial resolution of 0.31 cm/pixel. Similar to the processing of the MSIs, the RGB data resulted in a DSM at the original resolution of 0.31 cm/pixel, as seen in Figure 3c, and DTM at 1.55 cm/pixel in Figure 3d.

According to the methodology in [23], the canopy height model (CHM) can be simply estimated by calculating the difference between DSM and DTM layers as follows:

(1) CHM=DSM−DTM

To ensure the same dimension of the right two terms in Equation (1), we aggregated the DSM from the original resolution to that of DTM [24]. This allows us to implement the subtraction procedure between the DSM and DTM, resulting in the CHM at 8.35 cm/pixel and 1.55 cm/pixel from the MSI and RGB images, respectively.

Apart from the difference in spatial resolution, the DSM/DTM from the RGB data is about 2 m lower than those from the MSI data [25]. This result may be due to the performance of the GPS units in the sensors [26]. It also suggests the necessity of real-time kinematic (RTK) positioning and ground control points (GCPs) to improve the coordinate accuracy in the altitude dimension [27].

#### 2.2.2. Biomass Estimation

To develop the predictive model for the biomass, we calculated the NDVI and excess green (ExG) indices from the MSI and RGB data [28,29].

(2) ExG=2G−B−R

where G, B, and R correspond to the normalized relative reflectance in the RGB data. Different vegetation indices were compared in [30,31] to retrieve vegetation biomass from multispectral data, and the NDVI obtained be best performance. Other vegetation indices such as red-edge vegetation index and canopy chlorophyll content index can be useful when the NDVI is saturated in dense vegetation conditions [32]. However, for the alfalfa crops, the NDVI was not saturated due to relatively lower height and biomass. Furthermore, high collinearity was found among different indices using the MSI and RGB data [33]. As the objective of this study is to evaluate different sensors for the UAV system to monitor alfalfa height and biomass, we selected the most representative NDVI and ExG indices for the MSI and RGB data analysis, respectively. These two indices were analyzed in terms of ground-measured biomass. Then, we established statistical relationships between the vegetation indices and ground measurements recorded on 8 August 2022. These statistical models were then used to retrieve the biomass on 12 September 2022.

**Figure 4:**
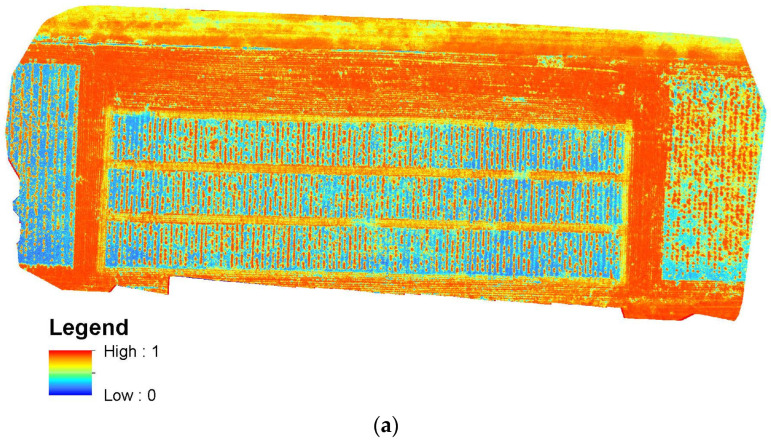

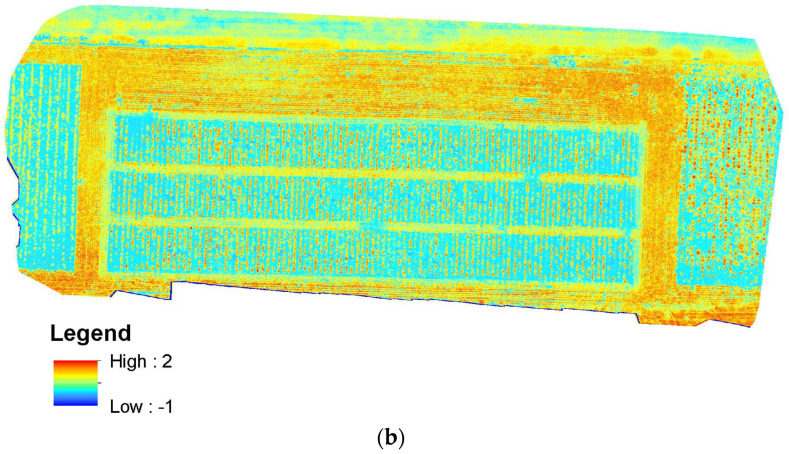
Spatial distribution of (**a**) MSI NDVI and (**b**) RGB ExG on 13 October 2022.

As shown in Figure 4a, the NDVI values were computed from MSI to characterize the plant growth dynamic and to characterize the biomass. Aklilu Tesfaye and Gessesse Awoke [34] reported the saturation issue of NDVI, but for the forage crop, the small fall height and biomass range may not reach the saturation threshold [35]. To compare the performances between MSI and RGB data to estimate the biomass, we computed the ExG in Figure 4b from the RGB data.

We recognized that NDVI and ExG are traditional vegetation indices to characterize crop growth dynamics. The objective of this paper is to suggest an optimal selection of a suitable sensor between MSI and RGB to characterize different crop growth parameters such as height, biomass yield, vegetation cover, and leaf area index (LAI). We used statistical metrics such as correlation coefficient (R), root mean square error (RMSE), and mean absolute error (MAE) to evaluate the performances of the proposed approaches.

## 3. Results and Discussions

### 3.1. Forage Canopy Height from MSI and RGB Data

**Figure 5:**
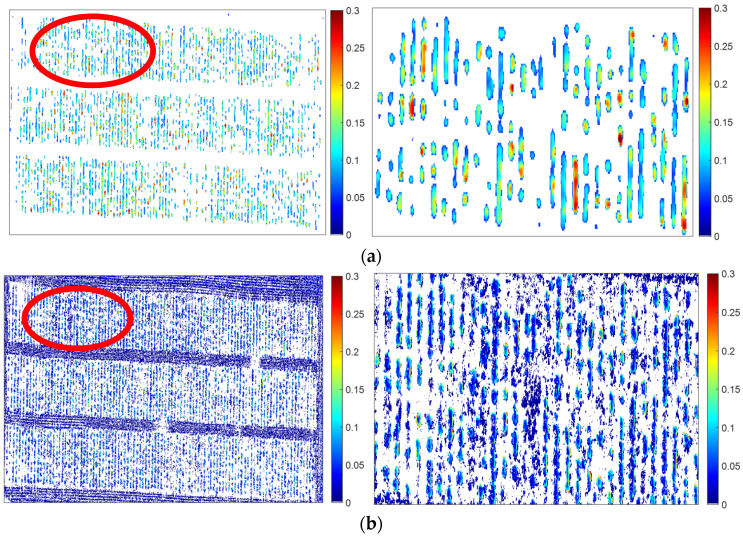
Canopy height model (CHM) from (**a**) MSI and (**b**) RGB imagery.

The CHM from both the MSI and RGB data in Figure 5 captured the spatial variability among different plots and within a single plot. This highlights the advantages of the UAV-based sensing technology to characterize plant growth at individual plots or even a single plant. The CHM from the MSI in Figure 5a is about 0.1 m higher than that from the RGB in Figure 5b. For a single plot, there are more valid retrievable pixels from RGB-derived CHM than from the MSI-derived CHM. More retrieved CHM information from RGB data suggests a potentially better result compared to the MSI.

The pixels of the canopy height model (CHM) results were averaged for each plot. The resulting plot-averaged height was compared with the ground measurements. In this study, by considering the range of ground measurements, we set two threshold criteria for the invalidity of the retrieved height component: height > maximum (60 cm) or height < minimum (5 cm, 10 cm). We consider the estimated values may contain more uncertainty if the height estimates are less than the minimum threshold.

**Figure 6:**
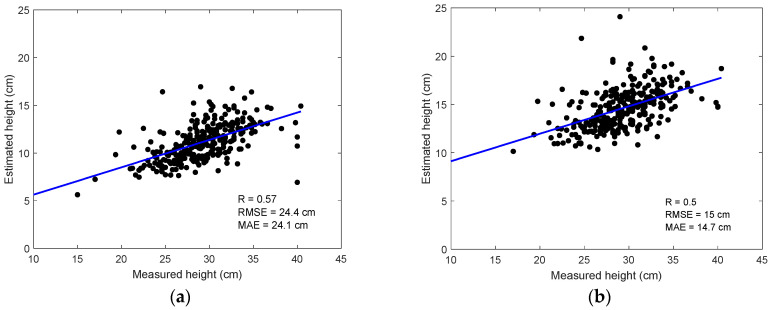
Validations of the retrieved height from MSI by setting the minimum threshold as (**a**) 5 cm and (**b**) 10 cm, in terms of ground measurements.

Figure 6 shows the height estimate from MSI with the same maximum threshold but different minimum thresholds. The threshold of 5 cm obtained a higher correlation than that of 10 cm. However, in both cases, underestimations were observed. Nevertheless, as the minimum threshold changed from 5 cm to 10 cm, the underestimations were mitigated. Compared to the 10 cm minimum threshold, we obtained more pixels within a single plot when the minimum threshold was set to 5 cm. In the next parts of this paper, we consider developing empirical equations to correct the bias in height estimation.

In Figure 6, the deviations between the retrieved and measured height may be partially due to the difference in the observation locations and measurement timing. The manual height measurements were based on the average scaling of the three samples for each plot, but the height retrieval using CHM used all available pixels covered by the corresponding plot. Thus, CHM represents pixel-based height at different locations over a plot. In this experiment, we did not acquire the pre-seeding baseline DEM on bare soil. Thus, the DTM data were generated from the spatial interpolation of soil pixels in the DSM, which led to the reduced spatial resolution of the DTM. For the very high-resolution CHM, we may need to acquire baseline RGB or MSI data over the pre-seeded soil conditions.

**Figure 7:**
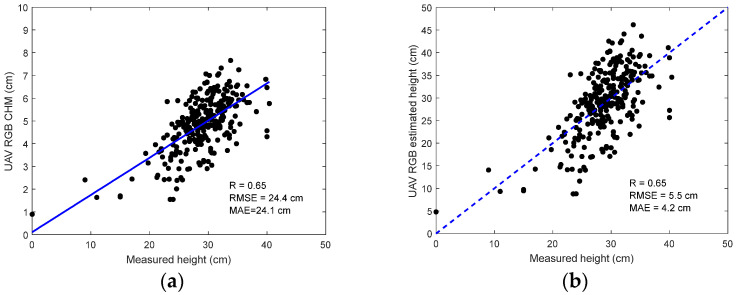
Validations of the retrieved height from RGB data: (**a**) original retrieval; (**b**) bias correction for height estimation.

In comparison, Figure 7a shows the estimated canopy height from the RGB data. In agreement with Figure 4, the estimates from the RGB were significantly lower than those from the MSI. In either case, the canopy height was underestimated. In order to correct the bias of the height (*H*) estimation, we developed a linear correction function as follows:

(3) H=(CHM−0.11)/0.16

By applying Equation (3) to the DSM estimate using RGB data, we obtained the final estimation of the height in Figure 7b. The bias-corrected height estimate agreed with the ground measurement (R = 0.65). However, the coefficients in Equation (3) need to be calibrated when applied to different scenarios.

### 3.2. Forage Above-Ground Biomass from MSI and RGB Data

As reported in [6,36], the NDVI is promising to monitor biomass changes over the crop growing period. In this study, we evaluated the NDVI results from the UAV MSI platform with a very high spatial resolution (1.67 cm). For each plot, the NDVI pixels covered were averaged and analyzed in terms of ground-measured fresh biomass (kg) per plot. The fresh biomass was measured two times on different periods and compared with the one-time point UAV image acquisition.

**Figure 8:**
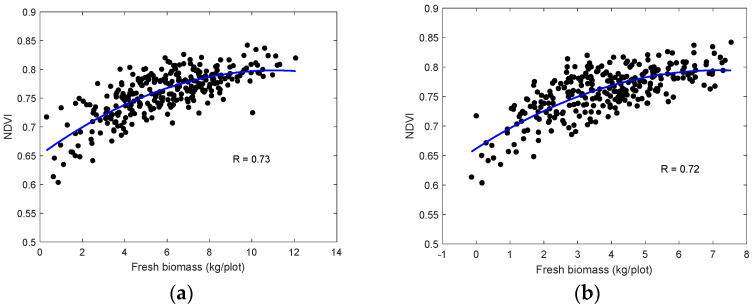
Relationships between UAV-based mean NDVI values for each plot on 13 October and measured fresh biomass on (**a**) 8 August and (**b**) 12 September 2022. Each plot was designed with an area of 6.27 m^2^.

As shown in Figure 8, the average NDVI values present strong sensitivity to the ground-measured fresh biomass on 8 August and 12 September, respectively. This allowed us to develop statistical modeling algorithms to accurately retrieve above-ground biomass from the UAV MSI images.

**Figure 9:**
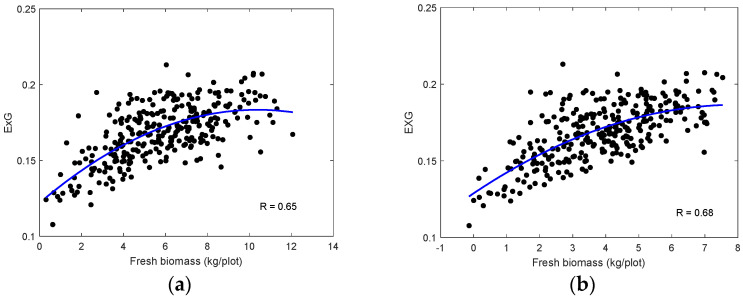
Relationships between UAV-based mean ExG values for each plot (black dots) on 13 October and measured fresh biomass on (**a**) 8 August and (**b**) 12 September 2022.

Similarly, we calculated the average values of the RGB ExG index and analyzed them in terms of ground measurements in Figure 9. Compared to the MSI, the sensitivity of ExG to ground measurements was reduced. Thus, the MSI data seem more robust for biomass estimation than the RGB data.

Based on the sensitivity analysis, the NDVI was selected to retrieve the biomass, while the ExG was not considered in the following. We used the biomass ground measurements on 8 August to develop the empirical relationships with the NDVI:

(4) $$B=79.28\times NDVI^{2}-74.15\times NDVI+16.33$$

**Figure 10:**
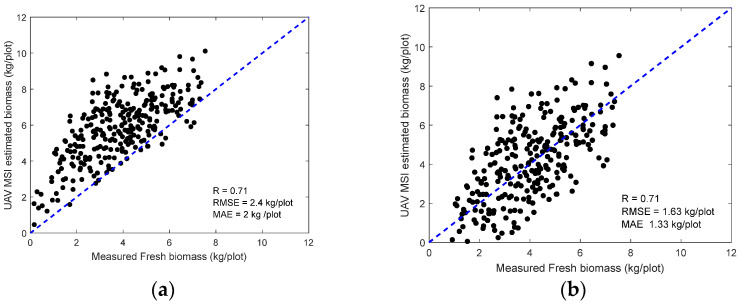
UAV multispectral-retrieved (**a**) biomass and (**b**) bias-corrected biomass on 12 September 2022.

The developed models between NDVI and biomass (*B*) were evaluated over the ground samples measurement on 12 September 2022. Figure 10a shows the comparison between the retrieved and ground-measured biomass, indicating a reasonable agreement (R = 0.71). Due to the limited number of UAV data acquisition, this result in Figure 10a was not an independent assessment of the model performance, but it suggests insights into the feasibility of the MSI imaging approach to estimate the forage biomass. The coefficients in Equation (4) need to be calibrated when applied to different sites.

Similar to the height estimation, we developed a bias correction function using data points presented in Figure 10a. After applying the bias correction, the ultimate estimated above-ground biomass (AGB) agrees with the ground measurement in Figure 10b.

(5) AGB=(B−2.98)/0.75

It should be mentioned that the coefficients in Equation (5) need to be recalibrated when used to different datasets over other experimental sites. This is the major limitation of the empirical models, compared to the physical models.

In this study, the biomass was measured per plot basis. Thus, the consistency in plot size may impact the statistical metrics. In further studies, we will consider standard biomass measurements over unit square meters.

## 4. Conclusions

This study proposes to monitor the forage crop (alfalfa) canopy height and above-ground biomass by comparing the performances between UAV-based MSI and RGB sensors.

The crop height was extracted from very high spatial resolution MSI and RGB images. Reasonable correlations were found between digitally derived canopy height and ground measurements (R = 0.50~0.65). When the height estimates were small, the uncertainty was found to be larger. Thus, it was necessary to set certain minimum thresholds in order to discard the invalid pixels. The RGB sensors obtained better retrieval performance for canopy height than the MSI sensor. However, the canopy height was significantly underestimated. To overcome this limitation, we developed bias-adjusted empirical equations in the canopy height estimation.

On the other hand, the traditional NDVI was found promising to characterize the fresh biomass yield (R = 0.73). This may be due to the relatively small size of the forage plant, which did not reach the saturation threshold found in the NDVI parameter. Compared to the ExG index from the RGB data, the NDVI from the MSI data had better performance in retrieving the above-ground biomass. This indicates the importance of the near-infrared wavelength to characterize the vegetation growth dynamics.

In this paper, we developed methods for physical parameter estimation using different UAV sensors. The UAV-based MSI and RGB imagery provided solutions to enhance precision agriculture and plant phenotyping. This study contributes to the selection of different sensors by considering the purpose of interesting crop-growing parameters such as height and biomass yield. The limitation of this study is the temporal gap between the UAV data acquisition and fresh biomass measurements. In perspective, we will acquire better UAV data and ground measurements with minimum temporal gaps to validate the statistical models in this paper. Furthermore, we plan to further investigate the potential of hyperspectral and LiDAR sensors to characterize the growth of different crop types.

## Data Availability

Data are contained within the article.
